# SGSM2 inhibits thyroid cancer progression by activating RAP1 and enhancing competitive RAS inhibition

**DOI:** 10.1038/s41419-022-04598-y

**Published:** 2022-03-09

**Authors:** Xi Su, Dong Chen, Lizhang Zhu, Hao Jia, Jiaxuan Cai, Peng Li, Bin Han, Donglai Wang, Hongtao Li, Jiaqian Fan, Mengwei Gu, Yaqi Zhou, Haixia Guan, Wei Wei

**Affiliations:** 1 Department of Thyroid and Parathyroid Surgery, Peking University Shenzhen Hospital, Shenzhen Peking University-The Hong Kong University of Science and Technology Medical Centre, Shenzhen, Guangdong Province China; 2 Center for Energy Metabolism and Reproduction, Shenzhen Institute of Advanced Technology, Chinese Academy of Sciences, Shenzhen, China; 3 Shenzhen College of Advanced Technology, University of Chinese Academy of Sciences, Shenzhen, China; 4 Department of Statistics and Data Science, Southern University of Science and Technology, Shenzhen, China; 5 School of Life and Health Sciences, Chinese University of Hong Kong, Shenzhen, Guangdong China; 6 Beijing Century Joyo Information Technology Co., Ltd, Shenzhen, China; 7 Department of Otorhinolaryngology, Peking University Shenzhen Hospital, ShenZhen Peking University-The Hong Kong University of Science and Technology Medical Centre, Shenzhen, China; 8 Department of Endocrinology, Guangdong Provincial People’s Hospital, Guangdong Academy of Medical Sciences, Guangzhou, Guangdong China

**Keywords:** Prognostic markers, Tumour biomarkers, Thyroid cancer

## Abstract

Thyroid cancer (TC) is one of the most common malignancies involving the head and neck, and its incidences are increasing every year. Small G protein signaling modulators 2 (SGSM2) belongs to a newly identified protein group that contributes to numerous cancer progression. However, its role in TC remains unknown. The aim of this study was to explore the functions and underlying molecular mechanism of SGSM2 in the progression of thyroid tumorigenesis. Here, we demonstrated that SGSM2 expression was markedly decreased in TC, and that lower SGSM2 expression was potentially related to worse patient prognosis. Meanwhile, the SGSM2 levels were not directly correlated with BRAF or RAS mutations in TC. Based on our functional analysis, ectopic SGSM2 expression strongly prevented cell proliferation, migration, invasion, and tumorigenic activity in TC cells that harbored wild type RAS. Mechanistically, we demonstrated that SGSM2 interacted with Small G protein Ras-associated protein 1(RAP1) and augmented its activity. Activated RAP1 then competitively suppressed RAS activation and thereby downregulated output of MAPK/ERK and PI3K/Akt networks, which are primary contributors of TC. In summary, the present study reports a tumor suppressive role of SGSM2 in TC. Moreover, we revealed the underlying molecular mechanism, thus providing a potential therapeutic target for TCs that harbor wild type RAS.

## Abstract (web summary)

A schematic model of SGSM2 suppressing the progression of RAS^WT^ thyroid cancer.

## Graphical abstract

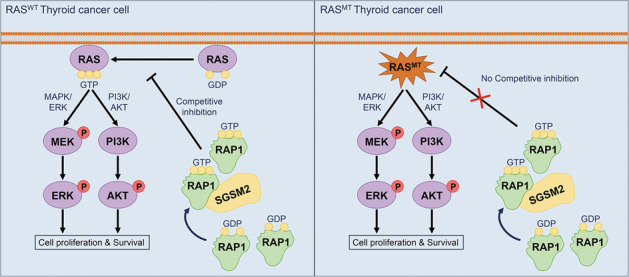

## Introduction

In recent years, thyroid cancer (TC) incidences have sharply risen all over the world, including China. Currently, it is the most prevalent malignancy in the head and neck [1–3]. Histologically, TC can be stratified into 4 categories: papillary TC (PTC, 80–85%); follicular TC (FTC, 10–15%); anaplastic TC (ATC, < 2%), which originates from the thyroid follicular epithelial cells; and medullary TC (MTC, 3–5%), which originates from unregulated replication of parafollicular C cells [4, 5]. Patients with PTC and FTCs usually have better prognosis. But, about 10% of cases can become more severe, with dedifferentiated forms of TC. TC with poor differentiation and anaplasticity tend to have very poor prognosis, and there are no effective treatment options [4, 6, 7]. Hence, there is a compelling need for developing new therapeutic targets and prognosis indicators for TC.

Excessive stimulation of Mitogen-activated protein kinase/extracellular signal-regulated protein kinase (MAPK/ERK) and phosphatidylinositol 3-kinase/Protein Kinase B (PI3K/AKT) networks in thyroid follicular epithelial cells induces the epithelial cell transformation into TC cells [4, 8]. Genetic alterations in members of the MAPK/ERK and PI3K/Akt networks, such as, BRAF and RAS mutations, can trigger aberrant activation of each pathway [9–11]. Moreover, alterations in cell adhesion [12] or gene promoter hypermethylation [13] are also implicated in tumor progression. The small G proteins, including RAS, are a group of low-molecular-weight GTPases that serve as molecular switches regulating cellular function [14, 15]. Aberrant small GTPase regulation often promote tumor progression via activation of different signal pathways, including MAPK/ERK and PI3K/Akt networks [16–18].

The novel protein group SGSM1/2/3 was first identified as regulators of certain small G protein-driven networks in 2007 [19]. Recent reports claimed that this protein group has complex functions in numerous cancers [20]. These proteins contain two major motifs: TBC and RUN. The TBC motif is present in a majority of GTPase-activating proteins, and it serves as a catalytic domain for GDP/GTP exchange and binding domain for the small G protein RAB [21]. The novel motif RAPID in the RUN-motif-neighboring region is crucial for the association and modulation of RAP proteins [19]. RAP proteins are known to hold critical functions in cancers. Being a RAP protein member, RAP1 competitively inhibits RAS activation [22–24]. SGSM2 proteins serve as GTPase activating proteins, and regulate G protein signaling via interaction with RAP1 [19, 25]. However, our goal was to determine the significance of SGSM2 in TC and identify the underlying mechanism.

## Results

### Low SGSM2 expression in PTC indicates poor prognosis

**Fig. 1:**
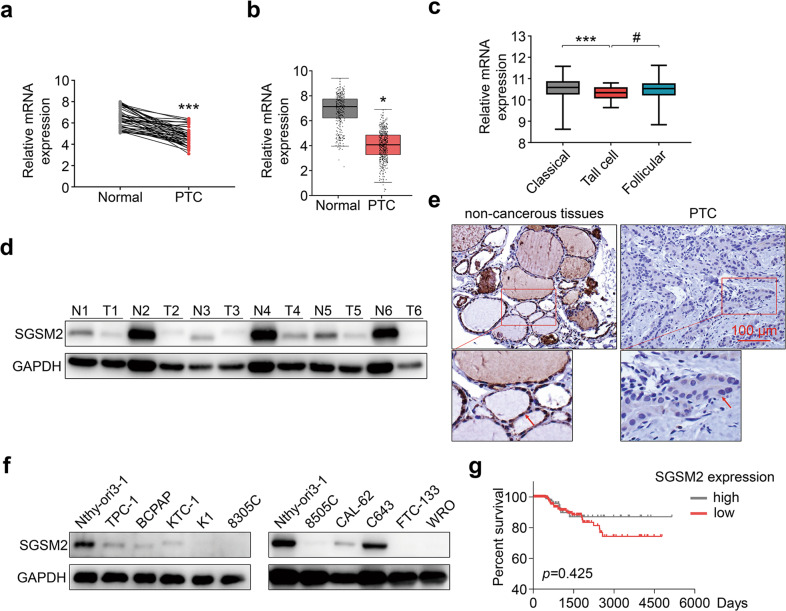
SGSM2 expression is decreased in primary thyroid cancer (TC). **a** Relative SGSM2 mRNA levels was lower in PTC samples, relative to normal thyroid tissue (NTT) samples, 18 S RNA was used for normalization of qRT-PCR data. **b** SGSM2 was lower in PTC, compared to matched NTT in the TCGA cohort. **c** Relative SGSM2 mRNA levels in different subtypes of PTC samples from the TCGA dataset, ***, compared to Classical PTC, #, compared to Follicular PTC. **d** SGSM2 expression in PTC and matched NTT samples, as evidenced by Western blot. **e** SGSM2 protein expression (indicated by the red arrow) in epithelial cell was lower in PTC, compared to adjacent NNT samples. **f** SGSM2 expression was examined in various TC cell lines and immortalized thyroid epithelial cells via Western blot, GAPDH employed as loading control. **g** Relationship between SGSM2 expression and patient survival in PTC, median SGSM2 expression was employed as threshold. Data shown as mean ± SD. ***P < 0.001; */#*P* < 0.05.

We analyzed SGSM2 expression in 46 PTC samples and paired normal thyroid tissues (NTT), using qRT-PCR. Based on our data, SGSM2 mRNA expression in PTC was significantly reduced, relative to NTT (Fig. 1a). We next assessed The Cancer Genome Atlas (TCGA) dataset and further confirmed the scarce SGSM2 expression in PTCs (Fig. 1b). Moreover, the SGSM2 expression was strongly associated with the histological type of PTC (Table 1). Based on our analysis, SGSM2 expression was much lower in tall-cell PTC (tcPTC), which is the most aggressive subtype of PTC [4], than both conventional PTC (CPTC), and follicular variants PTC (FVPTC) (Table 1 and Fig. 1c). Next, we performed Western blot and ICH analyses to determine SGSM2 protein expression in PTC samples and paired NTT samples. As shown in Fig. 1d, e, SGSM2 protein expression in PTC samples was also reduced, compared to the paired NTT. Consistently, elevated SGSM2 expression was observed in human immortalized thyroid epithelial cell Nthy-ori3-1, relative to TC cells. In addition, only two TC cell lines (CAL-62 and C643) that carry RAS mutation [26] exhibited some expression of SGSM2 (Fig. 1f). Furthermore, we also assessed the correlation between SGSM2 level and overall survival (OS) of PTC patients using the TCGA dataset and Kaplan–Meier survival curves, with the median expression as the threshold value. Based on our data, low SGSM2 expression showed some association with worse OS, particularly long-term OS, in PTC patients, but it did not reach significance (Fig. 1g). Given that BRAF and RAS mutations are highly prevalent in PTC [4], we further analyzed SGSM2 expression in patients with different genetic backgrounds using the TCGA datasets. However, no statistical difference was observed between the mutated and wild-type groups (Supplemental Fig. 1). Based on prior work demonstrating that combining BRAF mutation with other genetic alterations can be beneficial in predicting TC aggressiveness [6, 27], we analyzed the prognosis of patients carrying both BRAF mutation and low SGSM2 levels in the TCGA cohort. Our results indicated that the prevalence of both BRAF mutation and low SGSM2 expression can potentially predict worse prognosis than patients with PTC, although the difference was not significant (Supplemental Fig. 2). Collectively, these results indicate that SGSM2 serves a tumor suppressive role in TC.

**Table 1:** Correlation between SGSM2 expression and clinicopathological variables in patients with PTC (*n* = 501).

Variable	SGSM2 Expression (median as cutoff)				χ2-test
	High		Low		*P*-value
	Number	%	Number	%	
Age (years)					0.648
Mean	47.26		46.91		
SD	15.19		17.12		
Gender					0.978
Female	178	49.72	180	50.28	
Male	72	50.35	71	49.65	
Lymph metastasis					0.264
No	121	52.84	108	47.16	
Yes	129	47.43	143	52.57	
Histological type					0.007
Classical	196	53.55	170	46.45	
Tall cell	6	21.43	22	78.57	
Follicular	43	45.26	52	54.74	
Other	5	41.67	7	58.33	
Survival status					0.207
Death	5	31.25	11	68.75	
Alive	245	50.52	240	49.48	

### SGSM2 suppresses the malignant biological properties of TC cells carrying the wild type RAS (RAS^WT^)

**Fig. 2:**
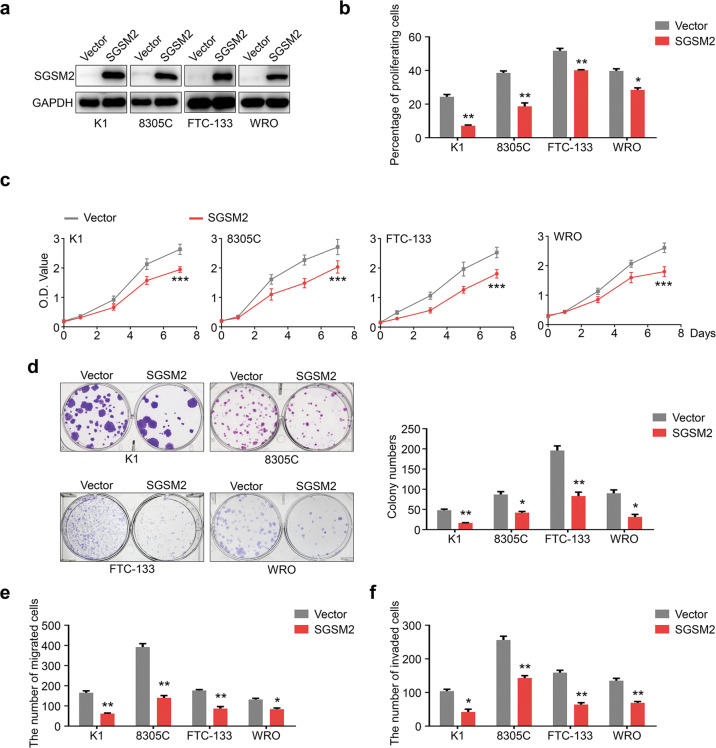
SGSM2 inhibits malignant biological properties of thyroid cancer (TC) cells. **a** Ectopic SGSM2 levels in different TC cells, as evidenced by Western blot. SGSM2 overexpression significantly inhibited TC cell proliferation, as evidenced by EdU assay **b** and MTT assay **c**. **d** SGSM2 overexpression markedly suppressed colony formation of TC cells. SGSM2 overexpression markedly reduced migration **c** and invasion **d** of TC cells. Data shown as mean ± SD. ***P < 0.001; **P < 0.01; *P < 0.05.

**Fig. 3:**
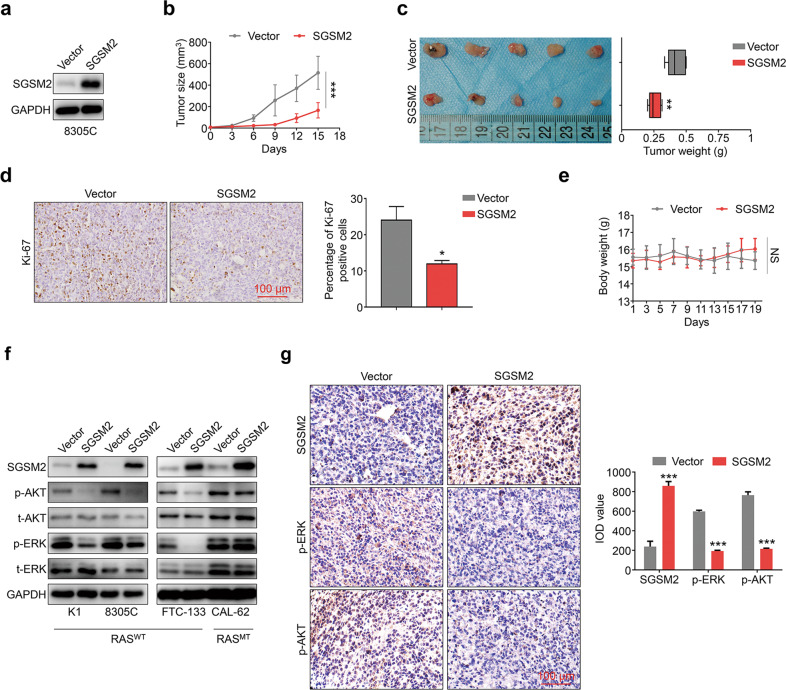
SGSM2 prevents tumor growth via suppression of both MAPK/ERK and PI3K/AKT networks in xenograft mouse models and in thyroid cancer (TC) cells. **a** Stably expressing SGSM2 in the 8305 C cell line, as evidenced by Western blot. **b** The growth curve of xenografts derived from the 8305 C stably expressing SGSM2 or non-sense control (NC). **c** Images of dissected tumors. Box-whisker plot illustrates average tumor weight. Data expressed as mean ± SD (*n* = 5/group). **d** A typical Ki-67 staining of a dissected xenograft tumor shown in the left panel, and quantification of Ki-67 positive cells presented in the right. **e** Alterations in nude mice body weight. **f** The levels phosphor-ERK (p-ERK), and phosphor-AKT (p-AKT) were decreased in cells overexpressing SGSM2 and carrying the wild type RAS. **g** Representative IHC staining depicting the reduced p-ERK and p-AKT levels in 8305 C cell-derived xenograft mouse model, and quantitative IOD values shown in the right. Data shown as mean ± SD. NS, not significant; *P < 0.05; **P < 0.01; ***P < 0.001.

To examine the biological activity of SGSM2 in TC, we ectopically expressed SGSM2 in four RAS^WT^TC cell lines, namely, K1, 8305 C, FTC-133, and WRO (Fig. 2a). We next performed the EdU assay to examine whether SGSM2 inhibits TC cell proliferation. As expected, SGSM2 overexpression decreased the percentage of proliferating cells in all examined cell lines (Fig. 2b & Supplemental Fig. 3). Next, we performed MTT assay to further verify our findings. As shown in Fig. 2c, ectopically expressed SGSM2 significantly suppressed TC cell proliferation. These data were further supported by colony-forming assay (Fig. 2d). Since the major cause of cancer recurrence is metastasis, we investigated whether SGSM2 expression affects migration and invasion of TC cells. Based on our data, SGSM2 overexpression markedly impaired TC cell migration, as opposed to the vector-treated cells (Fig. 2e & Supplemental Fig. 4a). In addition, ectopically expressed SGSM2 inhibited TC cell invasion (Fig. 2f and Supplemental Fig. 4b). Furthermore, we demonstrated that SGSM2 expression was strongly correlated with lymph metastasis in the TCGA dataset, using univariate analysis (Table 2). However, we observed that the SGSM2 expression does not influence the malignant biological properties of TC cells carrying the RAS mutant (RAS^MT^) (Supplemental Fig. 5a–e). These findings indicate that SGSM2 selectively suppresses the malignant biological properties of RAS^WT^ TC cells. To further validate the above results, we stably expressed SGSM2 in both RAS^WT^ TC line 8305 C (Fig. 3a) and RAS^MT^ TC line CAL-62 (Supplemental Fig. 6a), and established xenografts models of nude mice with screened cells. We revealed that SGSM2 overexpression limited xenograft tumor growth driven by 8305 C (Fig. 3b), while no influence was observed in tumors driven by CAL-62 (Supplemental Fig. 6b). Moreover, SGSM2 overexpression produced a marked decrease in volume and weight of the 8305 C cell-based tumors (Fig. 3c), and not the CAL-62 cell-based tumors (Supplemental Fig. 6c). These results also corroborated with the Ki-67 staining of the aforementioned tumors (Fig. 3d & Supplemental Fig. 6d). Apart from this, no obvious difference in body weight was seen in the different groups (Fig. 3e & Supplemental Fig. 6e). These results further support the tumor suppressive role of SGSM2 in RAS^WT^ TC.

**Table 2:** Relationship between metastasis and *SGSM2* expression testing by univariate and multivariate cox regression analysis (*n* = 501).

Variable	Univariate analysis		Multivariate analysis	
	Hazard ratio	*P*	Hazard ratio	*P*
Lymph metastasis	0.64 (0.41–0.98)	0.04^*^	0.62 (0.38–1.01)	0.06
Distant metastasis	0.73 (0.47–1.11)	0.14	0.65 (0.39–1.10)	0.11

### SGSM2 prevents TC growth via suppression of the MAPK/ERK and PI3K/AKT networks

The MAPK/ERK and PI3K/AKT networks drive TC development and malignancy progression, and are also the primary targets for anti-TC therapy [4, 28]. Accordingly, we explored whether SGSM2 overexpression regulates these two pathways. As shown in Fig. 3f, SGSM2 overexpression suppressed ERK and AKT phosphorylation in RAS^WT^TC cells. However, in RAS^MT^ TC cells, ERK and AKT phosphorylation remained unaltered, even though SGSM2 was successfully overexpressed (Fig. 3f). To validate the above observations in our mouse model, we first performed H&E staining of our tumor xenografts (Supplemental Fig. 7a, b). Next, IHC staining was used to detect SGSM2 levels, as well as ERK and AKT phosphorylation statuses in paraffin sections. We revealed that ERK and AKT phosphorylation was markedly reduced in 8305 C cell-based tumors, but not in CAL-62 cell-based tumors, upon successful SGSM2 overexpression (Fig. 3g & Supplemental Fig. 6g). Based on these results, SGSM2 serves as a tumor suppresser in RAS^WT^ TC cells via suppression of the MAPK/ERK and PI3K/AKT network activities.

### SGSM2 suppresses the MAPK/ERK and PI3K/AKT networks via activation of RAP1

**Fig. 4:**
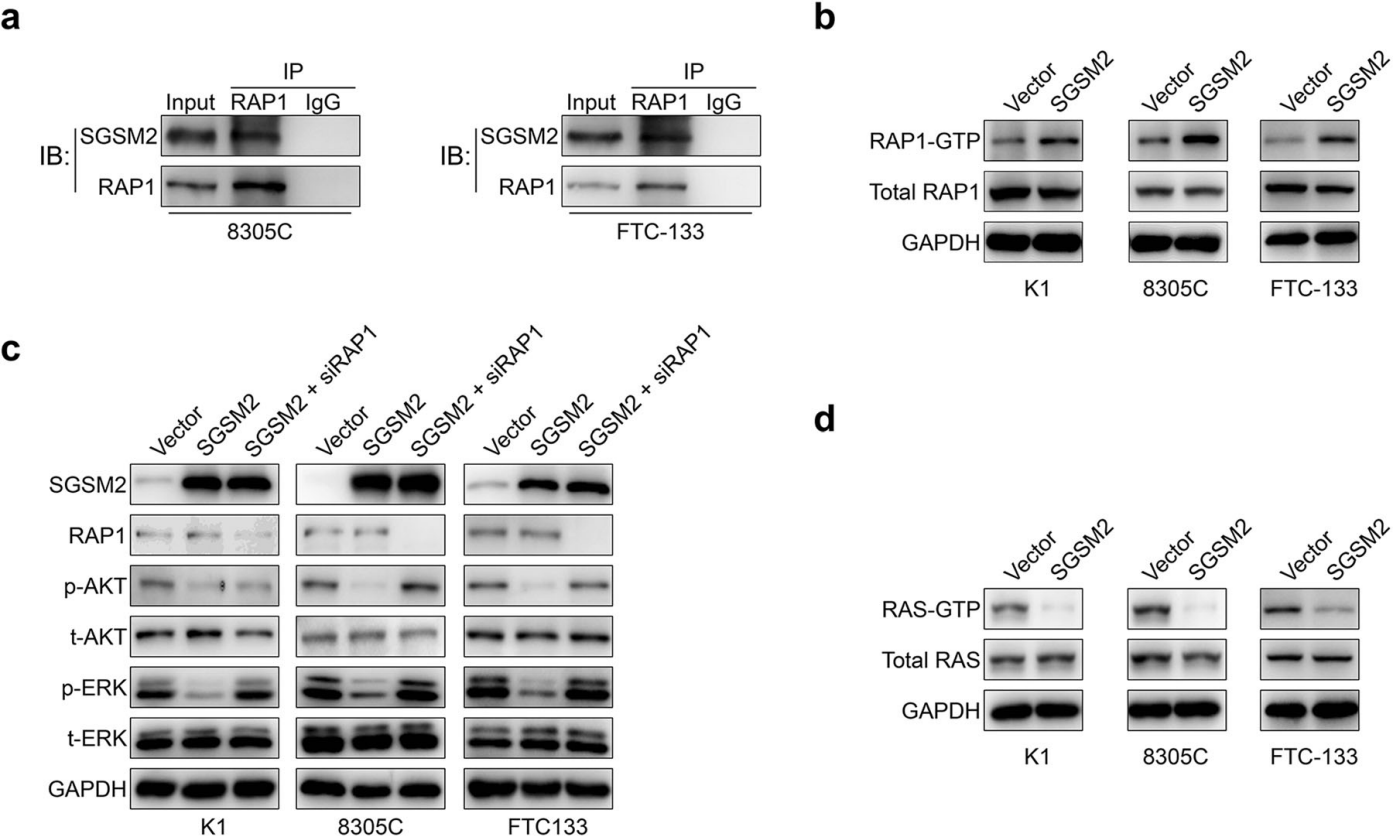
SGSM2 inhibits the MAPK/ERK and PI3K/AKT networks via activation of RAP1. **a** Lysates of 8305 C and FTC-133 overexpressing SGSM2 were immunoprecipitated with antibody against RAP1, and then immunoblotted with antibody targeting SGSM2 to confirm the intrinsic association between GMPS and TRIM21. **b** SGSM1 overexpression promoted RAP1 activation in indicated TC cells. Lysates of indicated cells were evaluated for RAP1 activation by detecting GTP-bound RAP1, and total RAP1 was determined using input samples. **c** Knock down RAP1 in K1, 8305 C, and FTC-133 cells restored p-ERK and p-AKT levels. **d** SGSM1 overexpression inhibited RAS activation in indicated TC cells. Lysates of indicated cells were employed for RAS activation assessment via evaluation of GTP-bound RAS levels, and total RAS levels were determined with input samples.

It was previously reported that SGSM family members modulate and interact with an important small G protein RAP1, which is a key mediator of RAS [24, 25]. Hence, we speculated that SGSM2 loss in TC suppresses RAP1, which, in turn, relieves the competitive inhibition of wild type RAS. Therefore, SGSM2 overexpression must suppress RAS and its downstream pathways. To verify our hypothesis, we first detected the interaction between SGSM2 and RAP1 by immunoprecipitation assay and proved the association between SGSM2 and RAP1 in both cell lines (Fig. 4a). Next, we detected the active form of RAP1 (GTP-liganded, RAP1-GTP) using RAP1 Activation Assay Kit. We revealed that SGSM2 overexpression in TC cells markedly enhanced RAP1-GTP levels (Fig. 4b). Moreover, reduced ERK and AKT phosphorylation, caused by SGSM2 overexpression, were rescued in RAP1 knock-down cells (Fig. 4c). These results indicated that SGSM2 inhibited both MAPK/ERK and PI3K/AKT networks via activation of RAP1. Thus, the mechanism of competitive RAS depression by RAP1 was revealed. We also detected the active form of RAS (GTP-liganded, RAS-GTP) using the RAS Activation Assay Kit. As expected, SGSM2 overexpression in TC cells markedly reduced RAS-GTP levels (Fig. 4d). These data suggest that SGSM2 promotes AP1 activity by competitively inhibiting wild type RAS activity.

## Discussion

The novel SGSM1/2/3 protein family members, distributed over in different tissues, were first identified as regulators of the small G protein-based signaling pathways in 2007 [19]. In recent years, the SGSM family was reported to serve as tumor suppressors during the formation and progression of numerous cancers [20, 29, 30]. In this study, we demonstrated that SGSM2 levels were markedly reduced in PTCs, compared to NTT. Although in our findings, lower SGSM2 levels were not significantly correlated with poor OS of PTC patients, we speculated that this is because of the long 5-year survival rate of PTC patients [31, 32]. However, we did reveal that reduced SGSM2 levels were related to lymph metastasis and the most aggressive subtype of PTC, tcPTC. Next, using a series of in vitro and in vivo functional analyses, we demonstrated that ectopic SGSM2 expression inhibited RAS^WT^ TC cell proliferation, colony formation, migration, invasion, and tumorigenic activity in nude mice. Collectively, based on our analysis, SGSM2 functions as a tumor suppressive gene in TC harboring the wild type RAS.

BRAF and RAS mutations are common in TC, and they cause numerous aberrant expressions of different TC-related genes [4, 10, 33, 34]. Combination of mutations and other molecular events, such as, TERT promoter mutations serves an essential function in predicting TC patient prognosis [6, 27]. Notably, in our study, low SGSM2 expression was not directly induced by the aforementioned mutations. This leads to the possibility that BRAF mutation prevalence and low SGSM2 expression may hold prognostic values for PTC patients. As expected, we observed that combining BRAF mutation and low SGSM2 expression produces a worse OS than respective simple genetic alterations in patients with PTC, though this was not significant. These results revealed the prognostic value of SGSM2 in TC. However, further clinical investigation, involving longer follow-up times, are warranted to confirm this assumption.

RAP1, one of the small G proteins, often switch between GTP-bound and—unbound states to activate downstream targets in order to convey biological messages and execute physiological functions [35, 36]. SGSM2 binds RAP1 with its special functional domain, and modulates its activation [19]. This is in accordance with our data that revealed that ectopic SGSM2 expression vastly increased GTP-bound RAP1. Aberrant RAP1 activation promotes different types of cancers via multiple signaling pathways, according to earlier studies [37–39]. However, our data indicated that RAP1 activation via ectopic SGSM2 expression inhibited TC progression by diminishing MAPK/ERK and PI3K/AKT network activities. This observation enlightens us on the complex RAP1 activities in various types of cancers. To further investigate the underlying mechanism, we tested the activity of RAS protein, which is upstream of the MAPK/ERK and PI3K/AKT networks in TC [4, 40]. Based on these evidences, RAP1 activation via ectopic SGSM2 expression reduces RAS activity, and inhibits the MAPK/ERK and PI3K/AKT networks. Our data is consistent with published reports that suggested that RAP1 may competitively suppress RAS activation, since they share a high sequence homology [41, 42], and that RAP1 may influence the RAS nanocluster [24].

The RAS proteins contain HRAS, KRAS, and NRAS that are highly homologous and have overlapping functions [43]. RAS proteins trigger numerous networks that regulate cell proliferation, differentiation, and survival [18]. Gain-of-function RAS mutations enhance GTP associations, owing to the rapid exchange of nucleotide and impaired GAP binding. This leads to it being insensitive to competitive inhibition [44, 45]. Our findings further supported that SGSM2 selectively suppressed the malignant biological properties of RAS^WT^ TC cells. Our data revealed that SGSM2 does not significantly influence the malignant biological properties of C643 (HRAS^MT^) and CAL-62 (KRAS^MT^) cells. We speculated that RAP1 activation does not induce competitive inhibition of mutant RAS. Nevertheless, additional mechanistic investigations are warranted to validate our hypothesis. Notably, BRAF is one of the downstream effectors of RAS, and BRAFV600E hyperactivity is independent of RAS [4, 46]. However, the PI3K/AKT network, downstream of RAS, is hardly affected by BRAFV600E [4]. This is consistent with our findings that SGSM2 diminished the output of both MAPK/ERK and PI3K/AKT networks and also inhibited the malignant biological properties of TC carrying BRAFV600E.

**Fig. 5:**
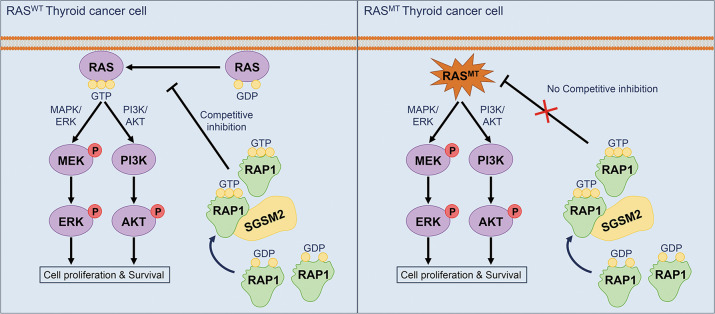
A schematic model of SGSM2 suppressing the progression of RAS^WT^ thyroid cancer (TC). SGSM2 binds and activates RAP1, and competitively inhibits the activities of wild type RAS. This diminishes the MAPK/ERK and PI3K/AKT networks in TC cells.

In summary, SGSM2 is decreased and serves a tumor suppressive role in RAS^WT^TC cells. Re-expressed SGSM2 limits malignant biological properties in these TC cells. SGSM2 binds and activates RAP1, thereby competitively inhibiting RAS activities in these TC cells. In this way, SGSM2 diminishes the MAPK/ERK and PI3K/AKT networks and inhibits progression of RAS^WT^TCs (Fig. 5). In general, our data demonstrates the tumor suppressive activity of SGSM2 in RAS^WT^ TCs, and provides an excellent prognostic and therapeutic candidate for this type of TC.

## Materials and methods

### Clinical samples and database

46 pairs of primary TC and NTT were acquired from the Peking University Shenzhen Hospital, and were approved by the Institutional Review Board and Human Ethics Committee of the Hospital. Patients’ characteristics are described in Supplemental Table 1. We received informed consent from all patients before initiation of the study. Patients included in this study did not undergo any treatment prior to study. All harvested tissues underwent histological assessment by two senior pathologists at the Department of Pathology at our hospital, based on the World Health Organization (WHO) guidelines. Paraffin-embedded sections were employed for immunohistochemical (IHC) analysis. Fresh samples were lysed for RNA and protein extraction. The expression and mutation data of each sample were taken from the TCGA Data Portal on 20 July 2020, and the patients’ characteristics were obtained from the TCGA data and are described in Supplemental Table 2.

### RNA extraction and quantitative RT-PCR (qRT-PCR)

RNA isolation, cDNA conversion, and qRT-PCR were conducted as reported before [47]. Relative RNA levels were computed using the 2^-ΔΔCt^ formula, and were adjusted to the *18* *S* rRNA expression. All mRNA values used in this study was mean of triplicates. Primer sequences are presented in Supplemental Table 3.

### IHC and H&E staining

Tumor tissues underwent paraffin embedding, prior to sectioning at 4 μm, followed by treatment with antibodies (Supplemental Table 4) at 1:200–300 dilution, and immunostaining, as instructed in a standard protocol, using the DAB Substrate Kit (ZSGB-BIO). Cell proliferation was determined by quantifying Ki-67 staining (percentage of positive cells), in the presence of an internal or intra-assay control. The Ki-67 protein levels received scores of 0, 1, 2, 3, which represented negative, weak positive, positive, and strong positive, respectively. Scores of 1, 2, 3 were considered positive, and the percentage of positive staining cells were determined. The protein levels were determined via integral optical density (IOD) using Image-pro plus 6.0 (Media Cybernetics, USA). We also stained xenograft tumors with hematoxylin and eosin (H&E), and assessed under the same magnification, light brightness, and exposure intensity.

### Western blot analysis

Cell lysis was done in prechilled RIPA buffer and protease inhibitors. The same amount of protein lysates were then separated via SDS–PAGE, prior to transfer on PVDF membranes (Roche Diagnostics), which were then treated with primary antibodies (Supplemental Table 4), with subsequent treatment with species-specific HRP-conjugated secondary antibodies from ZSGB-BIO, followed by visualization via a Western Bright ECL detection system (Tanon).

### Cell culture

TC cell lines K1, 8305 C, TPC-1, BCPAP, CAL-62, FTC-133, and human immortalized thyroid epithelial cells Nthy-ori3-1 were obtained with STR profiling from the Chinese Academy of Sciences, Shanghai Institute of Biochemistry and Cell Biology. 8505 C, C643, and WRO cells were provided by Prof. Haixia Guan. All cells were grown at 37 °C in RPMI 1640 medium (Gibco, Thermo Fisher Scientific) with 10% fetal bovine serum (Gibco, Thermo Fisher Scientific), 1% Non-Essential Amino Acids (Gibco, Thermo Fisher Scientific), and 1% Sodium Pyruvate (Gibco, Thermo Fisher Scientific). All cells were treated with a mycoplasma clearing reagent prior to experiments.

### Plasmid synthesis and transfection

SGSM2 (pcDNA3.1-SGSM2) plasmids were constructed, as described previously [47]. Cells were incorporated with specified plasmids at 70% confluence using the X-tremeGENE HP DNA Transfection Reagent (Catalog#06366244001. Roche Diagnostics GmbH, Mannheim, Germany).

### Transfection of short interfering RNAs (siRNAs)

The anti-SGSM2 (si-SGSM2-1and si-SGSM2-2) and anti-RAP1 siRNAs, as well as control siRNA (si-NC) were acquired from Gene Pharma (Shanghai, China). Cells were incorporated at 40% confluence with the X-tremeGENE siRNA Transfection Reagent (Catalog#04476093001, Roche Diagnostics GmbH, Mannheim, Germany) at a final siRNA concentration of 50 nM. The siRNA sequences are presented in Supplemental Table 3.

### Cell viability assay

Cells after gene knock down or overexpression were digested and seeded (2000/well) in 96-well plates. Following 0, 1, 3, 5, and 7 days of culture, 20 μl of 5 mg/mL 3-(4,5-Dimethylthiazolyl-2)-2,5-diphenyltetrazolium bromide (MTT) was introduced to cells, followed by an additional incubation at 37 °C for 4 h, at which point, the reaction was terminated with 150 μl of DMSO. Cell viability was then assessed using a microplate reader at 570 nm test wavelength and 670 nm reference wavelength. All MTT assays were performed thrice. The IC50 values were computed using the Reed-Muench formula [48].

### Cell proliferation assay

The 5-Ethynyl-2′-deoxyuridine (EdU) assay kits were purchased from Solarbio, Beijing (Cat # CA1170). Cells after gene knockdown or overexpression were digested and seeded (2 × 10^5^/well) in 12-well plates. Following a 24-h culture, the medium was altered to a medium with 50 μM EdU, and cultured for additional 2 h. Next, cells received fixation in 4% paraformaldehyde, prior to PBS rinse, and staining with Hoechst33342. Staining was assessed under a fluorescent inverted microscope (Leica, Wetzlar, Germany, Cat # DMI8), and color mergence was conducted with the ImageJ software (ImageJ version 1.44p, NIH, MD). Percentage of positive cells was determined from five arbitrary fields of view.

### Colony formation assay

To assess colony formation, we grew a monolayer of TC cells in a 6-well plate using a cell density of 2000–3000 cells per well. The medium was altered every 3 days. Following a 10–14 day of culture, colonies received fixation in 4% paraformaldehyde, prior to PBS rinse, and staining with a crystal violet solution. All assays were done thrice.

### Migration and invasion assay

Cells (1 × 10^5^) were overnight serum-starved, then plated into the top chamber of the transwell (8.0 μm pore size; Millipore, MA) plate in 200 μl of medium with 0.5% FBS. Both transwell chambers had Matrigel pre-coating (4× dilution; 15 μl/well; BD Bioscience, NJ) for invasion assay. A total 1 ml medium with 20% FBS was introduced to the bottom chamber. Upon a 14–20 h incubation period, the cells that remained at the top chamber were removed with a cotton swab, and the cells that migrated to the bottom chamber underwent fixation in 100% methanol, prior to staining with crystal violet solution (0.5% crystal violet in 2% ethanol). Assessment was done using 5 images captured arbitrarily from each membrane. The quantity of cells that migrated are presented as the mean quantity of cells of five arbitrarily selected fields of view.

### Animal studies

The lentivirus-based CRISPR-Cas9 technique (Shanghai GeneChem Co., LTD) was performed to stably knock in SGSM2 or control in 8305 C (5 mice per group) and CAL-62 (3 mice per group) cells. Next, cells (4 × 10^6^) were administered subcutaneously into the right armpit of 5-week-old female nude mice acquired from the SLAC laboratory Animal Co., Ltd. (Shanghai, China) to generate xenograft mice model. The xenograft mice were arbitrarily separated into 2 groups, without blinding. Tumor size was determined every two days beginning at 5 days post administration. The tumor volume was computed as follows: Tumor volume = length × width^2^ × 0.5. Mice were euthanized 20 days post administration and tumors were weighted and harvested for analysis. Our animal protocol received approval from the Institutional Review Board of ShenZhen Peking University-The Hong Kong University of Science and Technology Medical Centre.

### Immunoprecipitation (IP), RAP1 and RAS activities

Protein concentrations were normalized with equal incorporation prior to IP assay. Lysates were treated with specified antibodies or IgG at 4 °C for 4–5 h, with subsequent overnight treatment with protein A/G agarose beads (Catalog#: sc-2003, Santa Cruz, CA, USA) at 4 °C. The IP products were rinsed and analyzed via western blot. All IP and immunoblotting (IB) antibodies employed in IP assays were from various sources. A detailed antibody information is provided in Supplemental Table 2. Activated RAP1 and RAS were determined using the RAP1 (Sigma-Aldrich, cat:17-321) and RAS Activation Assay Kits (Sigma-Aldrich, cat:17-218), respectively.

### Statistical analysis

All data analyses employed the SPSS statistical package (16.0, SPSS Inc. Chicago, IL). Inter-group analysis was done with the unpaired student’s *t* test. Multi-group analysis was done with one-way analysis of variance (ANOVA) and subsequent Bonferroni’s multiple comparison test or two-way ANOVA with Bonferroni post-test. The Kaplan–Meier survival curve assessed PTC patient OS. Chi square test examined relationship between SGSM2 levels and patient clinicopathological features, with the SGSM2 median expression as the threshold. Uni- and multivariate analyses were employed to test the correlation between SGSM2 levels and metastasis. All data are shown as mean ± standard deviation (SD). *p* < 0.05 was regarded as the significance threshold. All experimentation was done in triplicates.

### Reporting summary

Further information on research design is available in the Nature Research Reporting Summary linked to this article.

## Supplementary information

Reporting Summary

Supplemental tables and figures

## Data Availability

Wei Wei and Xi Su have open access to all data and are responsible for the decision to publish this work. We declare the materials in this manuscript, including relevant data, to be freely available to any scientist wishing to use them, upon informing Wei Wei and Xi Su.
