# Supplementary material for: SGSM2 inhibits thyroid cancer progression by activating RAP1 and enhancing competitive RAS inhibition
Source: Cell Death Dis. 2022 Mar 9;13(3):218. doi: 10.1038/s41419-022-04598-y (PMC8907342; doi:10.1038/s41419-022-04598-y)
Supplement: Supplementary file 2 — Supplemental tables and figures [file 41419_2022_4598_MOESM2_ESM.docx]

**Supplemental Information**

**SGSM2 inhibits thyroid cancer progression via enhancing the competitive inhibition of RAS by RAP1**

Xi Su^1^, Dong Chen^1^, Lizhang Zhu^1^, Hao Jia^1^, Jiaxuan Cai^2,3^, Peng Li^1^, Bin Han^1^, Donglai Wang^1^, Hongtao Li^4^, Jiaqian Fan^5^, Mengwei Gu^6^, Yaqi Zhou^7^, Haixia Guan^8^*, Wei Wei^1^*

^1^Department of Thyroid and Parathyroid Surgery, Peking University Shenzhen Hospital, ShenZhen Peking University-The Hong Kong University of Science and Technology Medical Centre, Shenzhen, 518036, China.

^2^Center for Energy Metabolism and Reproduction, Shenzhen Institute of Advanced Technology, Chinese Academy of Sciences, Shenzhen, 518055, China.

^3^Shenzhen College of Advanced Technology, University of Chinese Academy of Sciences, Shenzhen, 518055, China.

^4^Department of Statistics and Data Science, Southern University of Science and Technology, Shenzhen, 518055, China.

^5^School of Life and Health Sciences, Chinese University of Hong Kong, Shenzhen, Guangdong, 518172, China.

^6^Beijing Century Joyo Information Technology Co., Ltd. Shenzhen Branch.

^7^Department of Otorhinolaryngology, Peking University Shenzhen Hospital, ShenZhen Peking University-The Hong Kong University of Science and Technology Medical Centre, Shenzhen, 518036, China.

8 Department of Endocrinology, Guangdong Provincial People's Hospital, Guangdong Academy of Medical Sciences, Guangzhou, Guangdong 510080, China.

**Conflict of Interest**

The authors declare that they have no conflict of interest.

***To whom correspondence should be addressed:**

Wei Wei M.D.

Department of Thyroid and Parathyroid Surgery, Peking University Shenzhen Hospital. Shenzhen, 518036, China.

No.112, LianHua Road, FuTian district, Shenzhen, 518036, China.

Tel/Fax: +86 13600405191; E-mail: [rxwei1123@163.com](mailto:ydm_723@163.com);

Haixia Guan M.D. and Ph.D.

Department of Endocrinology, Guangdong Provincial People's Hospital, Guangdong Academy of Medical Sciences. Guangzhou, 510080, China.

106 Zhongshan Er Road, Guangzhou, Guangdong 510080, China.

Tel/Fax: +86 18602491031; Email: hxguan@vip.126.com

**Supplemental Data**

**Supplemental Table 1**

Clinicopathological characteristics of the PTC patients (n=46)

**Supplemental Table 2**

Clinicopathological characteristics of the PTC patients in TCGA (n=501)

**Supplemental Table 3**

Sequences used in this study

**Supplemental Table 4**

The antibodies used in this study

**Supplemental Fig. 1**

Relative mRNA expression of SGSM2 in PTC samples with different BRAF or RAS mutation status.

**Supplemental Fig.2**

The prevalence of BRAF mutation and low SGSM2 expression potentially predicts the worse prognosis than respective effect in patients with PTC, the median expression was used as the cutoff point.

**Supplemental Fig.3**

Representative figures showed the proliferating cells in indicating cell lines.

**Supplemental Fig.4**

Representative figures showed the migrated and invaded cells in indicating cell lines.

**Supplemental Fig.5**

Knocking down of SGSM2 in C643 and overexpression SGSM2 in CAL-62 thyroid cancer cells don not affect their malignant biological properties.

**Supplemental Fig.6**

Stably expressing SGSM2 in CAL-62 cell line don not affect its malignant biological properties *in vivo*.

**Supplemental Fig.7**

H&E staining of the tumors in xenografts.

**Supplementary Table1. Clinicopathological characteristics of the PTC patients (n=46)**

| **Characteristics** | **No.** | **Percent** |
| --- | --- | --- |
| Gender |  |  |
| Male | 17 | 36.9 |
| Female | 29 | 63.1 |
| Age, years |  |  |
| Mean | 41.3 |  |
| SD | 5.8 |  |
| Disease stage |  |  |
| I | 43 | 93.5 |
| II | 3 | 6.5 |
| III | 0 | 0 |
| IV | 0 | 0 |
| lymphatic metastasis |  |  |
| No | 20 | 43.5 |
| Yes | 26 | 56.5 |
| Distant Metastasis |  |  |
| No | 46 | 100 |
| Yes | 0 | 0 |
| Survival status |  |  |
| Death | 0 | 0 |
| Alive | 46 | 100 |

**Supplementary Table2. Clinicopathological characteristics of the patients with PTC (n=501)**

| **Characteristics** | **No.** | **Percent** |
| --- | --- | --- |
| Gender |  |  |
| Male | 143 | 28.5 |
| Female | 358 | 71.5 |
| Age, years |  |  |
| Mean | 47.1 |  |
| SD | 15.9 |  |
| Disease stage |  |  |
| I | 282 | 56.3 |
| II | 58 | 11.6 |
| III | 111 | 22.1 |
| IV | 50 | 10.0 |
| Histological type |  |  |
| Thyroid Papillary Carcinoma- Classical/usual | 366 | 73.1 |
| Thyroid Papillary Carcinoma-Tall Cell (>= 50% tall cell features) | 28 | 5.6 |
| Thyroid Papillary Carcinoma-Follicular (>= 99% follicular patterned) | 95 | 18.9 |
| Other specify | 12 | 2.4 |
| lymphatic metastasis |  |  |
| No | 229 | 45.7 |
| Yes | 272 | 54.3 |
| Distant Metastasis |  |  |
| No | 489 | 97.6 |
| Yes | 12 | 2.4 |
| Survival status |  |  |
| Death | 16 | 3.2 |
| Alive | 485 | 96.8 |

**Supplementary Table 3.** Sequences used in this study

|  | **5’- Sequence -3’** |
| --- | --- |
| *SGSM2* primer-F | TCACCAAGGACGTGTGGAGCAA |
| *SGSM2* primer-R | TCCTTGCGGATCTCGTGCTCTA |
| *18S* primer-F | CGCCGCTAGAGGTGAAATTC |
| *18S* primer-R | CTTTCGCTCTGGTCCGTCTT |
| *Si-SGSM2-1* target sequence | TCCGATGAAAGACGCTGGT |
| *Si-SGSM2-2* target sequence | CCTGCACCGCATAGACAAG |

F: forward；R: reverse

**Supplementary Table 4.** The antibodies used in this study

| **Antibodies** | **Catalog#** | **Source** |
| --- | --- | --- |
| anti-RAP1 | Ab14404 | Abcam |
| anti-Ki67 | 550609 | BD Pharmingen |
| anti-t-AKT | BS1810 | Bioworld Technology |
| anti- p-AKT | BS4009 | Bioworld Technology |
| anti-GAPDH | AP0063 | Bioworld Technology |
| anti-t-ERK | #4695 | Cell Signaling Technology |
| anti-p-ERK1/2 | #4370 | Cell Signaling Technology |
| anti-RAP1 | #4938 | Cell Signaling Technology |
| anti-SGSM2 | NBP1-93637 | Novus Biologicals |

**
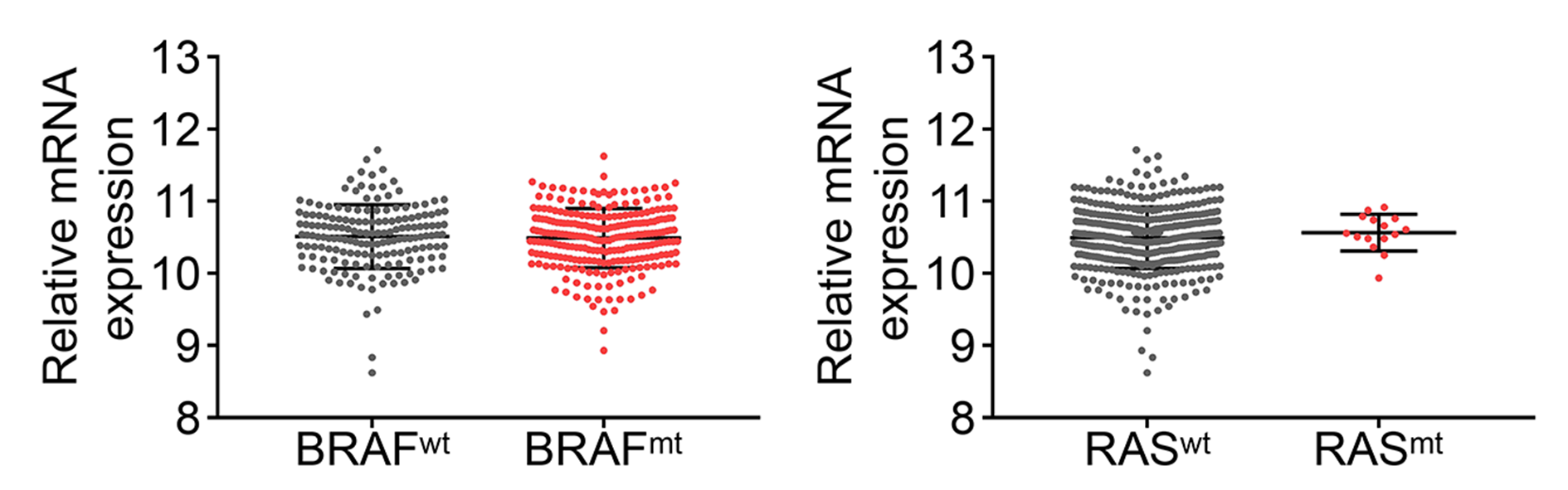
**

**Supplemental Fig.1** Relative mRNA expression of SGSM2 in PTC samples with different BRAF (left) or RAS (right) mutation status.

**
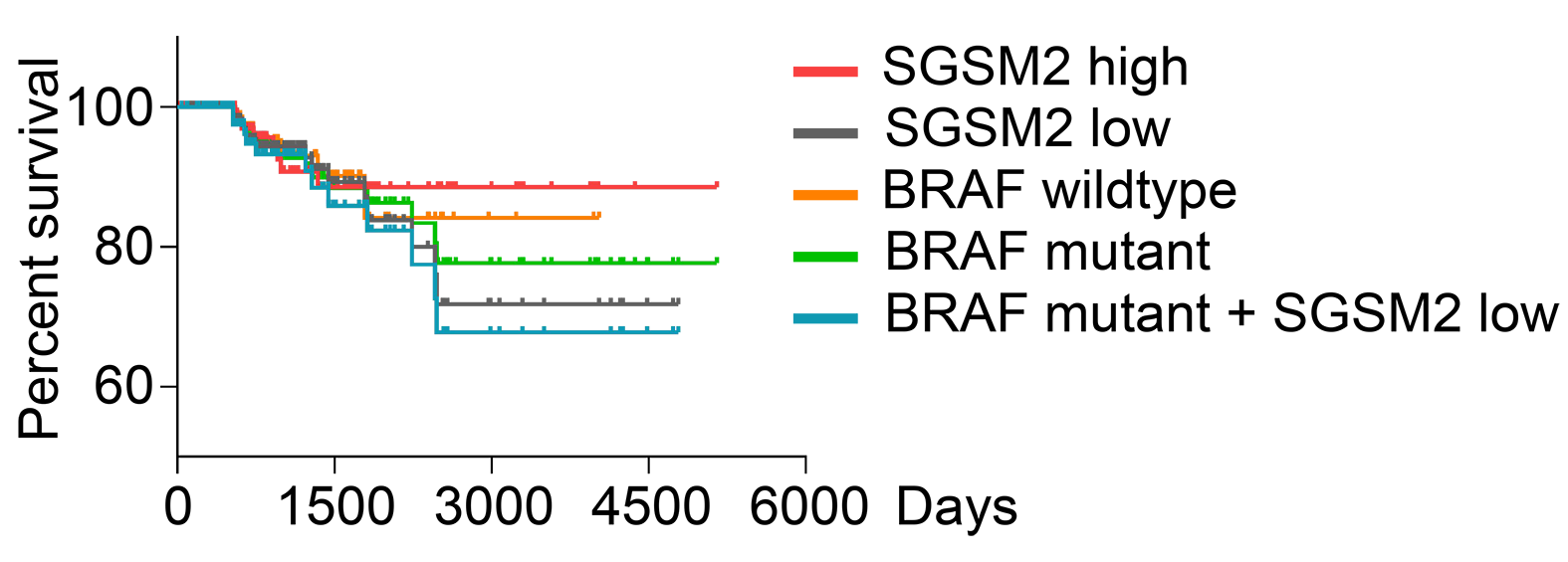
**

**Supplemental Fig.2** The prevalence of BRAF mutation and low SGSM2 expression potentially predicts the worse prognosis than respective effect in patients with PTC, the median expression was used as the cutoff point.

**
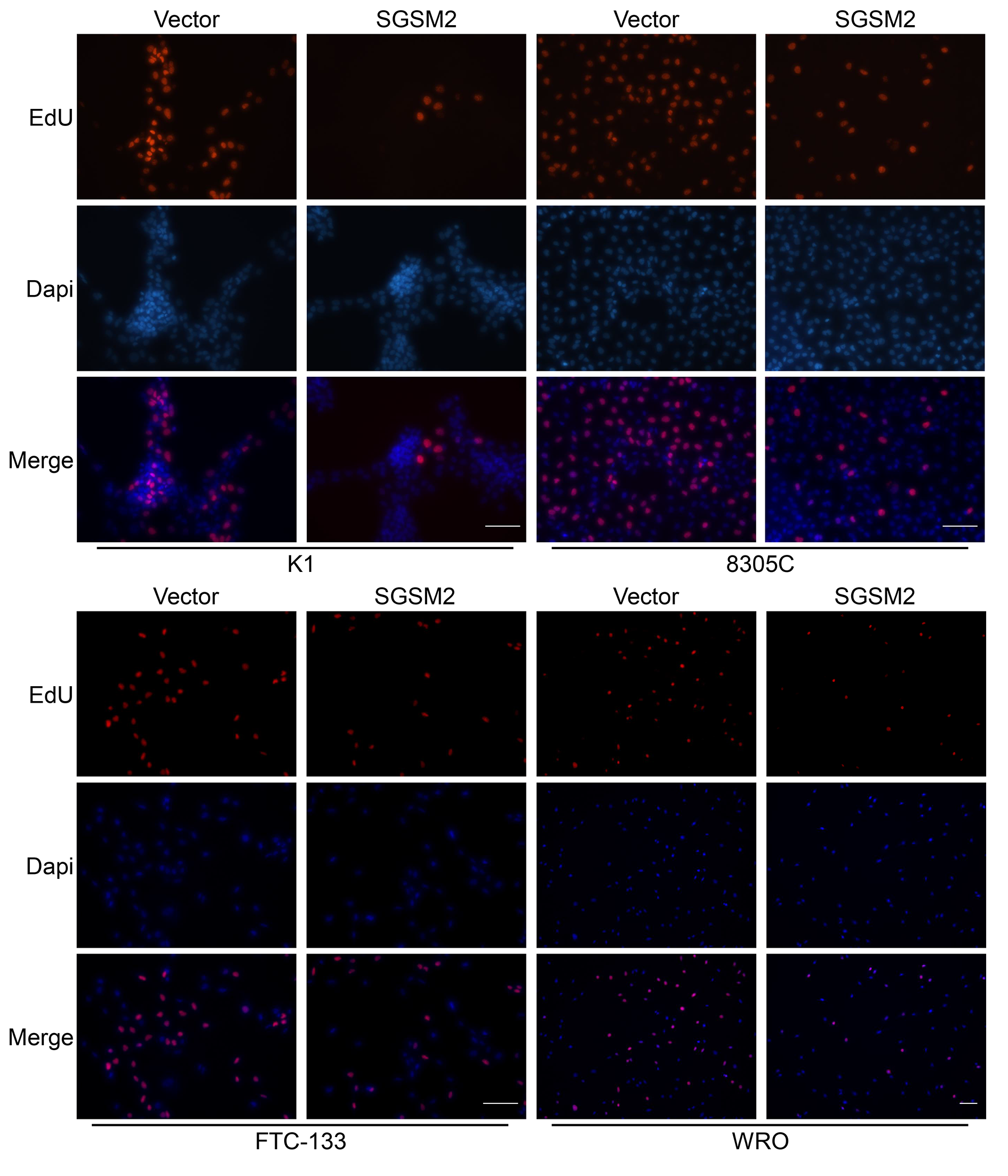
**

**Supplemental Fig.3** Representative figures showed the proliferating cells in indicating cell lines. Scale bar, 100 μm.

**
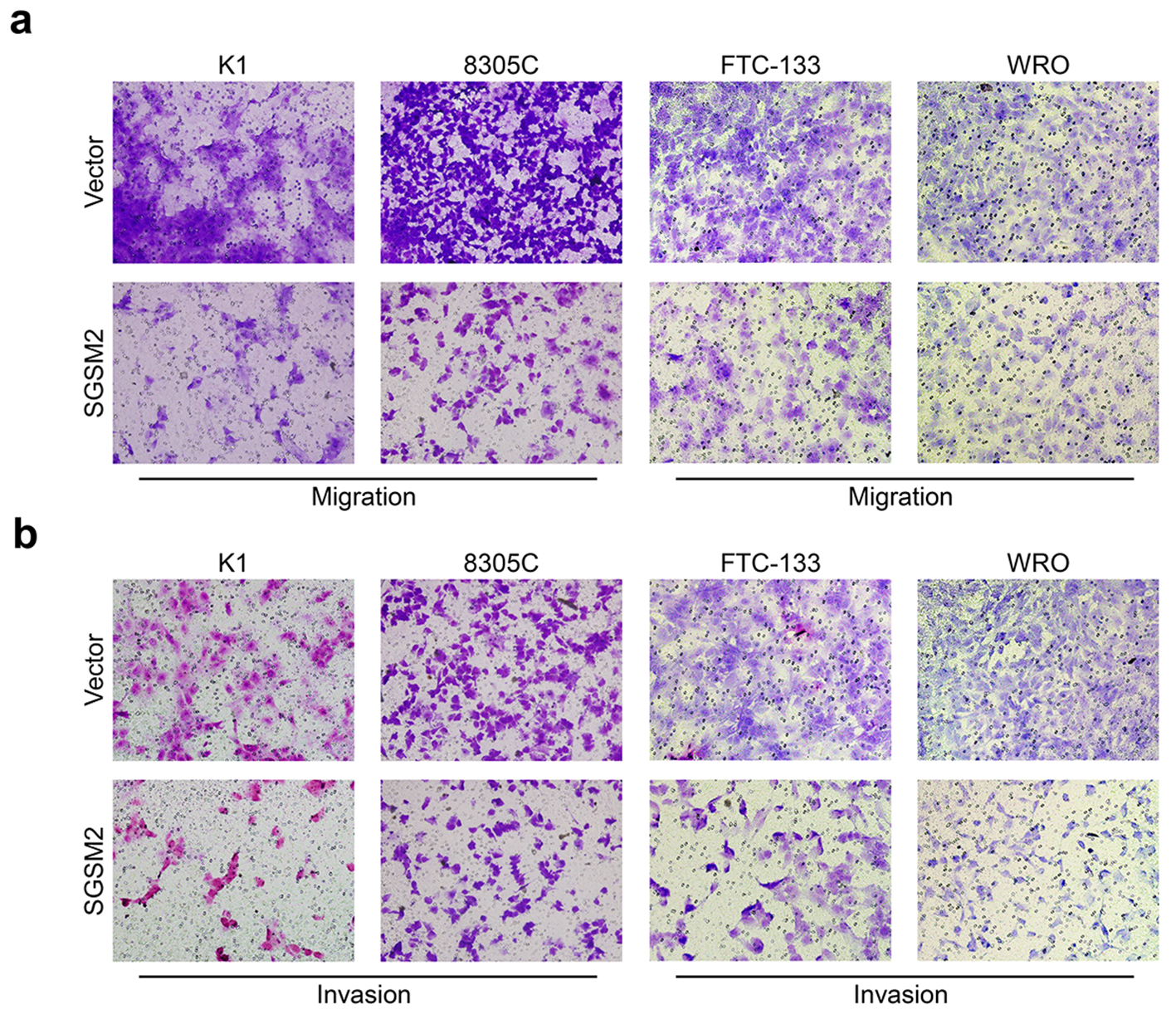
**

**Supplemental Fig.4** Representative figures showed the migrated **a** and invaded **b** cells in the chamber (200 times).

**
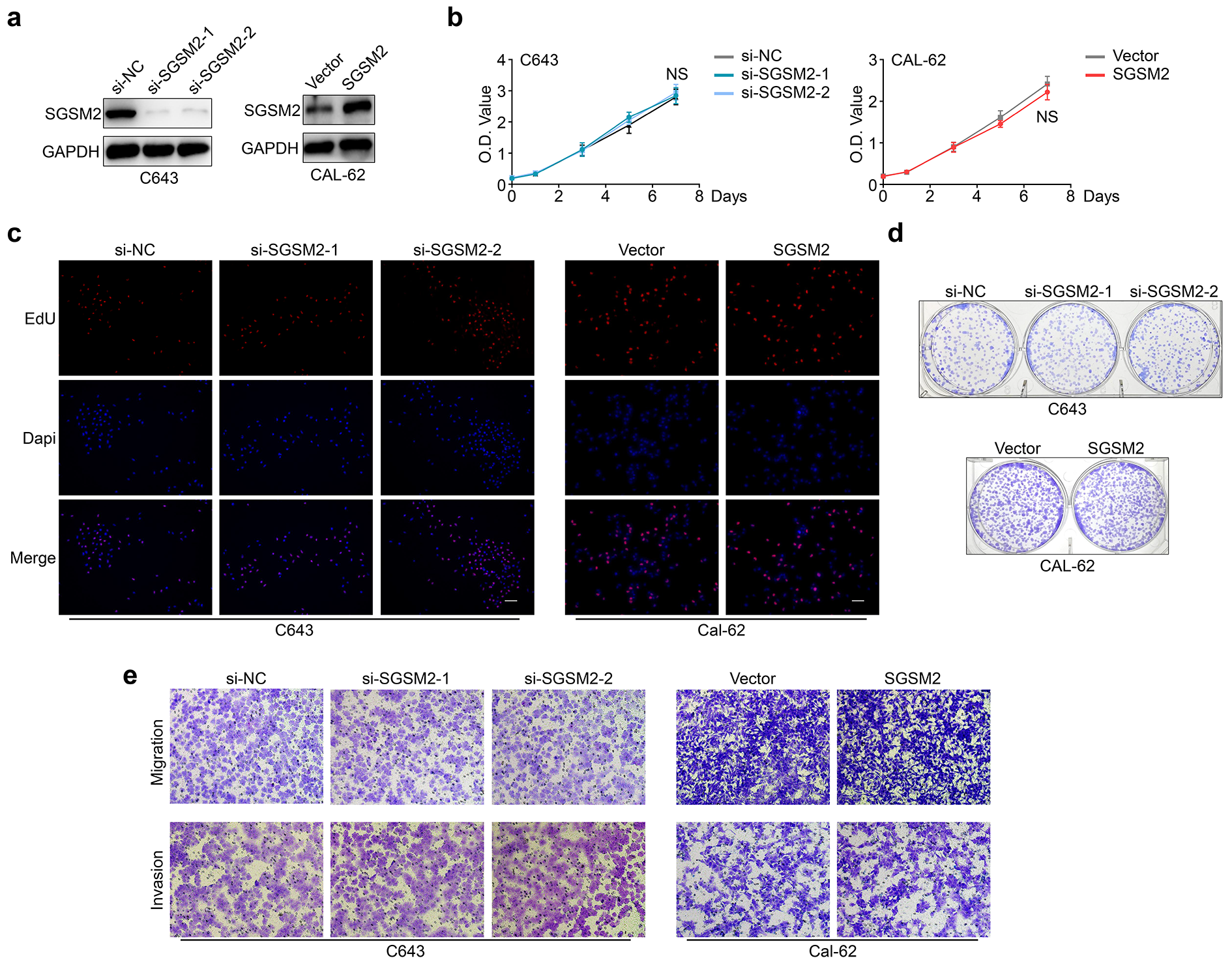
**

**Supplemental Fig.5 a** Knocking down and ectopic expression of SGSM2 in C643 and CAL-62 thyroid cancer cells respectively was determined by Western blot. The proliferation of indicating thyroid cancer cells were determined by MTT assay **b** and EdU assay **c**. **d** The colony formation of the indicating thyroid cancer cells. **e** The migration and invasion of indicating thyroid cancer cells. Data were presented as mean ± SD. NS, not significantly. Scale bar, 100 μm.

**
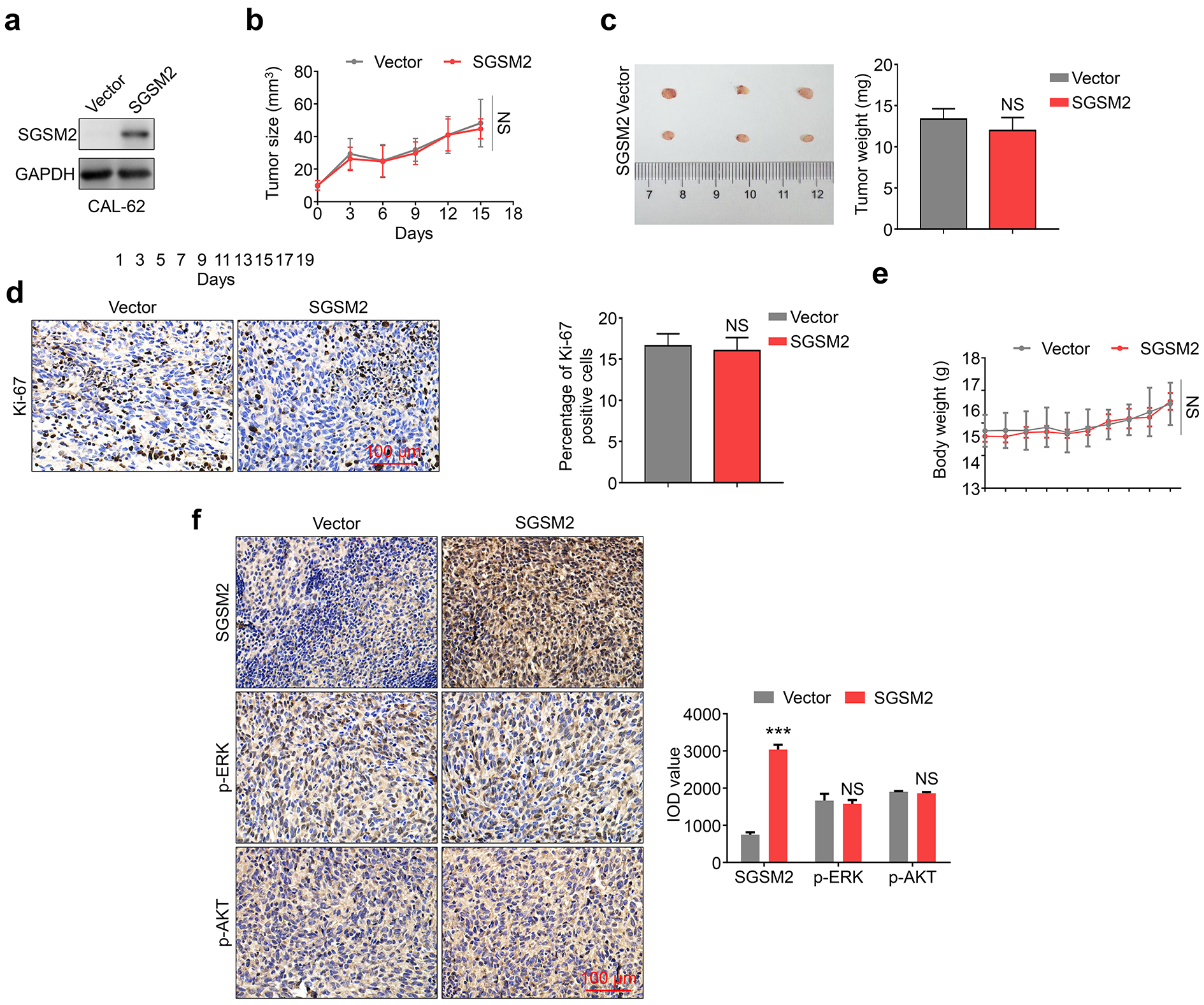
**

**Supplemental Fig.6 a** Stably expressing SGSM2 in CAL-62 cell line was determined by Western blot. **b** The growth curve of CAL-62 cell-derived xenografts stably expressing SGSM2 or non-sense control (NC). **c** Photographs of dissected tumors from the indicated groups. Box-whisker plot represents mean tumor weight in different groups. Data were shown as mean ± SD (n = 3/group). **d** Representative Ki-67 staining in dissected tumors from xenograft was shown in left panel, and quantitative percentage of Ki-67 positive cells was shown in right panel. **e** The body weight change of the nude mice. **f** Representative IHC staining show the level of SGSM2, p-ERK and p-AKT in CAL-62 cell-derived xenograft mouse model**,** and quantitative IOD value was shown in right panel. Data were presented as mean ± SD. NS, not significantly; ***, P <0.001.

**
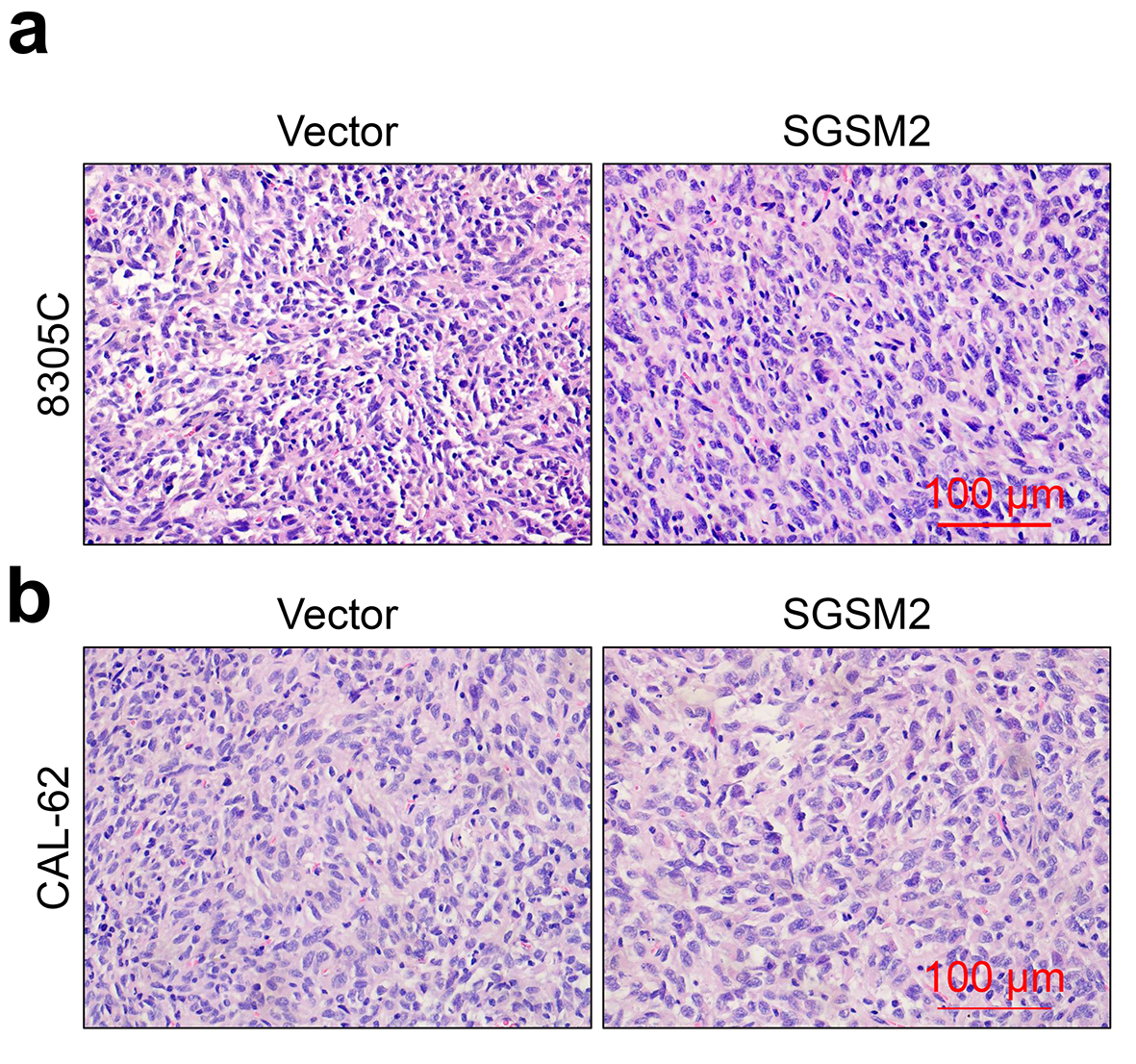
**

**Supplemental Fig.7 a** H&E staining of the tumors in 8305C cell-derived xenograft mouse model. **b** H&E staining of the tumors in CAL-62 cell-derived xenograft mouse model.
